# Clinical study of electroacupuncture on the recovery of gastrointestinal dysfunction after laparoscopic surgery for gastrointestinal cancer - study protocol for a randomized controlled trial

**DOI:** 10.1186/s12906-024-04418-0

**Published:** 2024-03-12

**Authors:** Shuet Ling Chung, Wen Li, Qiuyue Wang, Xiaofeng Qiu, Yuncheng Tang, Sheng Hu, Huangan Wu, Zhu Jin

**Affiliations:** 1 Department of Acupuncture, Moxibustion and Tuina, Shanghai Seventh People’s Hospital of Shanghai University of Traditional Chinese Medicine, Medicine. No 358, Da Tong Road, Gaoqiao Town, Pudong New District, Shanghai, 200137 China; 2 Department of Gastrointestinal Surgery, Shanghai Seventh People’s Hospital of Shanghai University of Traditional Chinese Medicine, Shanghai, China; 3 Shanghai Research Institute of Acupuncture and Meridian, Shanghai, China

**Keywords:** Electroacupuncture, Gastrointestinal surgery, Enhanced recovery after surgery, Gastrointestinal dysfunction, Laparoscopic surgery

## Abstract

**Background:**

Gastrointestinal dysfunction is one of the common complaints for patient post-surgery. Acupuncture has been employed to improve gastrointestinal function and sleeping quality and has confirmed clinical efficacy for emotional problems. This study aims to evaluate the clinical effect of electroacupuncture for postoperative rapid recovery.

**Methods:**

This study design is a two-arm, parallel, double-blinded randomized controlled trial. 104 subjects, aged from 40 to 89 years old, diagnosed with gastrointestinal cancer undergoing laparoscopic surgery, will be divided into Interventional Group and Control Group. Patients of both groups receive perioperative care under the guidance of ERAS guidance. The Interventional Group receives electroacupuncture treatment starting from the first day post-surgery for a consecutive 5 days, whereas the Control Group receives placebo electroacupuncture treatment. The primary outcome will be the first flatus time whereas the secondary outcomes will be the first sign of borborygmus, recovery of defecation, laboratory tests and questionnaires including Self-rating anxiety scale, Ford Insomnia Response to Stress Test, TCM rating scale of Gastrointestinal symptoms and Gastrointestinal Symptoms Rating Scales.

**Discussion:**

This study aims to provide timely intervention for post-laparoscopic patients with gastrointestinal tumour using the ERAS concept combined with electroacupuncture, observe the efficacy of this therapy in treating PGID, and contribute reliable scientific evidence for postoperative rapid recovery.

**Trial registration:**

Chictr.org.cn Identifier: ChiCTR2300078710. Registered on 15th December 2023.

**Supplementary Information:**

The online version contains supplementary material available at 10.1186/s12906-024-04418-0.

## Introduction

According to the Global Cancer Statistics for 2020, colorectal cancer is ranked as the third most commonly diagnosed cancer, and gastric cancer holds the fifth position. The morbidity and mortality rates for both have been steadily increasing, highlighting the substantial global burden they impose [1]. Thus far, surgical intervention remains the predominant solution for cancers in their early stages or without metastasis. While among surgical plans, laparoscopic surgery has the advantages of less trauma and faster recovery [2], which has gradually taken over traditional open surgery. However, the effect of both surgical plans remains controversial due to several factors. A meta-analysis related to the effect of complications of laparoscopic versus open surgery showed that there is no significant difference between both surgical plans in terms of infection [3]. In fact, laparoscopic surgery may still lead to post-surgery complications ranging from prolonged ileus, constipation to vomiting and nausea due to the usage of intraoperative anaesthesia drugs, carbon dioxide insufflation, etc [4].

The ERAS concept aims to improve the quality of life for patients by alleviating perioperative stress responses, reducing postoperative complications, shortening hospital stays, and relieving negative emotions. Its clinical pathway spans the entire diagnostic and treatment process, including pre-hospitalization, preoperative, intraoperative, postoperative, and post-discharge phases [5]. Based on previous studies, the adoption of the ERAS concept can significantly enhance the efficiency of surgical patient care, accelerate patient recovery, and improve postoperative quality of life [6–8]. In recent years, exploring other adjuvant treatment measures has become a hot topic and focus in clinical research. Many clinicians have actively incorporated acupuncture and electroacupuncture therapy based on the ERAS concept, aiming to achieve better therapeutic effects in promoting post-gastrointestinal surgery recovery. However, the use of the Eight Confluent Points remains a blank area in the rapid recovery after laparoscopic surgery for gastrointestinal tumours, representing an innovative approach both domestically and internationally.

A research progress on the molecular mechanism showed that electroacupuncture may relieve gastrointestinal mucosal damage and adjust gastrointestinal motility disorders via modulating the central and peripheral nerve signalling [9], while a study by Dr. Qiufa Ma’s team has proved the distal effect of acupuncture [10, 11]. Apart from that, inflammatory cytokines could predict and reflect the infectious complications of colorectal surgery where acupuncture therapy is likewise proved to be able to down-regulate serum levels of relative inflammatory factors of patients with cancer-related fatigue [12, 13]. Electroacupuncture as a TCM therapy, has improving effects on gastrointestinal dysfunction [14–16]. Among the clinical trials, acupoints Zusanli (ST 36), Tianshu (ST25), and Shangjuxu (ST 37) are frequently used. Therefore, the therapeutic role of electroacupuncture accelerating post-surgery gastrointestinal recovery using the Eight Confluent Points is elucidated in this trial, aiming to provide evidence for clinical use.

This study aims to provide timely intervention for post-laparoscopic patients with gastrointestinal tumour using the ERAS concept combined with electroacupuncture, observe the efficacy of this therapy in treating PGID.

## Materials and methods

### Study design

This study design is a two-arm, parallel, double-blinded randomized controlled trial aimed to study the effectiveness of electroacupuncture for improving the first flatus time of post-surgery patients. The study protocol followed the SPIRIT guidelines [17].

### Participants

#### Recruitment strategies

Participants will be recruited from the inpatient department of Gastrointestinal Surgery, Shanghai Seventh People’s Hospital. Recruitment posters will be distributed at the outpatient departments. Participants are considered for inclusion if they meet the criteria as follows.

#### Inclusion criteria

Patients eligible for the study must comply with the following criteria:

1. Diagnosed as primary gastrointestinal tumours including gastric, colon and rectal tumours; Undergoing laparoscopic surgery performed by the surgeons of the Gastrointestinal Surgery Department, Shanghai Seventh People’s Hospital;
2. TNM staging, Stage I-III;
3. Aged 40–89 years old (including 40 and 89 years old);
4. Consent to the participation of this study and is able to sign the informed consent form.

#### Exclusion criteria

Participants meeting any of the following criteria will be excluded:

1. Secondary gastrointestinal tumours;
2. Patients with cognitive dysfunction, unable to understand or fill assessment forms;
3. Pregnancy or lactation;
4. Severe illness with gastrointestinal system before surgery; Severe heart, lung, liver, kidney, brain or systemic immune diseases;
5. Allergic or intolerant to acupuncture.

#### Elimination criteria

1. Participants would be eliminated if they were found not meeting the inclusion criteria during the treatment period or statistical period.

#### Dropout criteria

1. Participants with poor adherence;
2. Participants fail to complete therapy within the requirement period;
3. Participants request to leave the study for any reason.

#### Discontinuation criteria

1. Participants request to leave the study;
2. Severe complications or infection occur post-surgery, in which the patient is to be transferred to another department for further specialized treatment;
3. Severe allergy or adverse events occur during the interventional period.

### Sample size

This randomized controlled trial aims to explore the improvement in the first flatus time of post-surgery patients on electroacupuncture therapy compared to placebo therapy. Based on a previous study conducted in China [18], sample size was calculated with 80% power and two-tailed α = 0.05, β = 0.20, taking the ratio of 1:1 for the interventional group and control group. Assuming a dropout rate of 15%, this study requires 52 subjects in each group, with a total of 104 subjects to be recruited.

### Ethics and trial registration

Ethics for this study protocol and informed consent materials have been approved by the Ethics Committee of Shanghai Seventh People’s Hospital affiliated to Shanghai University of Traditional Chinese Medicine on 5th December 2023, with committee approval number: 2023-7th-HIRB-078. This study has likewise been registered at Chinese Clinical Trial Registry, with approval number: ChiCTR2300078710.

### Procedure

**Fig. 1:**
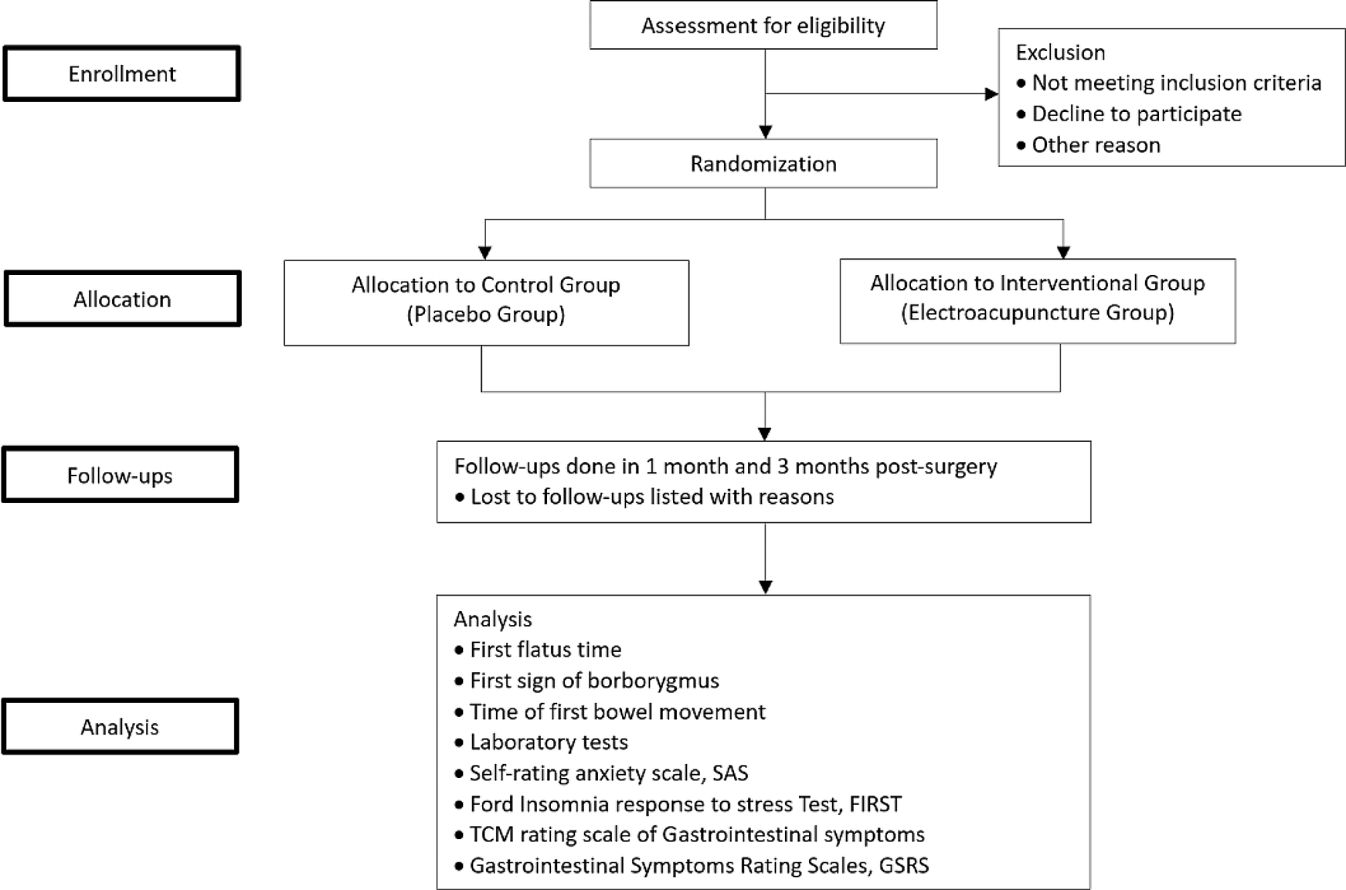
CONSORT flow diagram of study design

Participants will be assessed for eligibility by the doctors and nurses from the Gastrointestinal Surgery department before surgery. Participants who meet the criteria will be obtained informed consent. Then, the participants will undergo randomization and allocation before being enrolled in the treatment. Assessments and laboratory tests will be made according to the timeline (Fig. 1).

After the intervention period, the participants will receive postoperative care according to the guidelines of the Gastrointestinal Surgery department until they are discharged from hospital. Participants in the CG group will be invited to receive 5 acupuncture treatments free of charge on voluntary basis as post-trial care.

Follow-ups will be done on the first month and third month after the surgery.

### Randomization

The experiment adopts a simple randomization method for grouping, wherein a random number table is generated using SPSS 25.0 software. The random allocation cards are sealed in opaque envelopes and kept by designated personnel of the research team. When eligible subjects agree to participate in the trial, envelopes are assigned based on the order of patient visits. Only at this point can the envelope be opened, and the subjects are then enrolled in the treatment according to the assigned group.

### Blinding

According to the principle of blinding, group allocation information will be withheld from the subjects, data collection team and the statisticians. The subjects of both groups are blinded with the subjects in the control group receive placebo electroacupuncture therapy. Due to the profession of acupuncture techniques and the particularity of acupoints, the acupuncturists could not be blinded. They will be requested to perform the treatment according to the allocation and record any adverse events but will not assess the efficacy of the treatment. The data collection team is blinded. The team members will not be informed of the group allocation and will only be responsible for collecting data from the subjects. The statisticians are blinded. They are solely responsible for the statistical analysis and data interpretation. Unblinding will be taken if any serious adverse events happen, including serious complications caused by surgeries.

### Interventions

The electroacupuncture treatment and placebo treatment are designed based on expert consensus [19]. The intervention will be conducted by appointed licensed acupuncturists of Shanghai Seventh People’s Hospital, each with a minimum of 5 years of clinical experience in acupuncture. They are required to receive standardized training prior to trial. This study followed STRICTA [20] guidelines:

#### Interventional group – electroacupuncture group (EG)

Participants in the EG will receive electroacupuncture therapy every day for 5 days consecutively, from the first day post-surgery onwards, each session lasts 20 min. Single-use acupuncture needles (diameter 0.30 mm, length 40 mm, China Suzhou Medical Appliance Company 20162200970, Hwato) will be inserted on acupoints Neiguan(PC6), Gongsun(SP4) bilaterally, 4 acupoints in total (Table 1), after the sites of insertion are disinfected. Acupuncturists shall then perform gentle manipulations on all acupoints until muscle twitch responses are sought from participants. Next, paired alligator clips of the electroacupuncture apparatus (China Suzhou Medical Appliance Company 20172200710, Hwato) are attached at bilateral acupoints (PC6 to SP4, lengthwise), with a continuous wave of 10 Hz and a current intensity of 0.5 mA to 2 mA, depending on the patient’s tolerance level. The location of acupoints is referenced from “Nomenclature and Location of Acupuncture Points” (GB/T 12346 − 2021).

**Table 1:** Locations of Neiguan (PC 6) and Gongsun (SP 4)

Acupoints	Locations	Depth of insertion
Neiguan (PC 6)	On the palmar aspect of the forearm, 2 cun above the transverse crease of the wrist, between the tendons of m. palmaris longus and m. flexor carpi radialis.	15–30 mm, vertically
Gongsun (SP4)	On the medial aspect of the foot, in the depression distal and inferior to the base of the first metatarsal bone	15–25 mm, vertically

#### Control group – placebo group (PG)

**Fig. 2:**
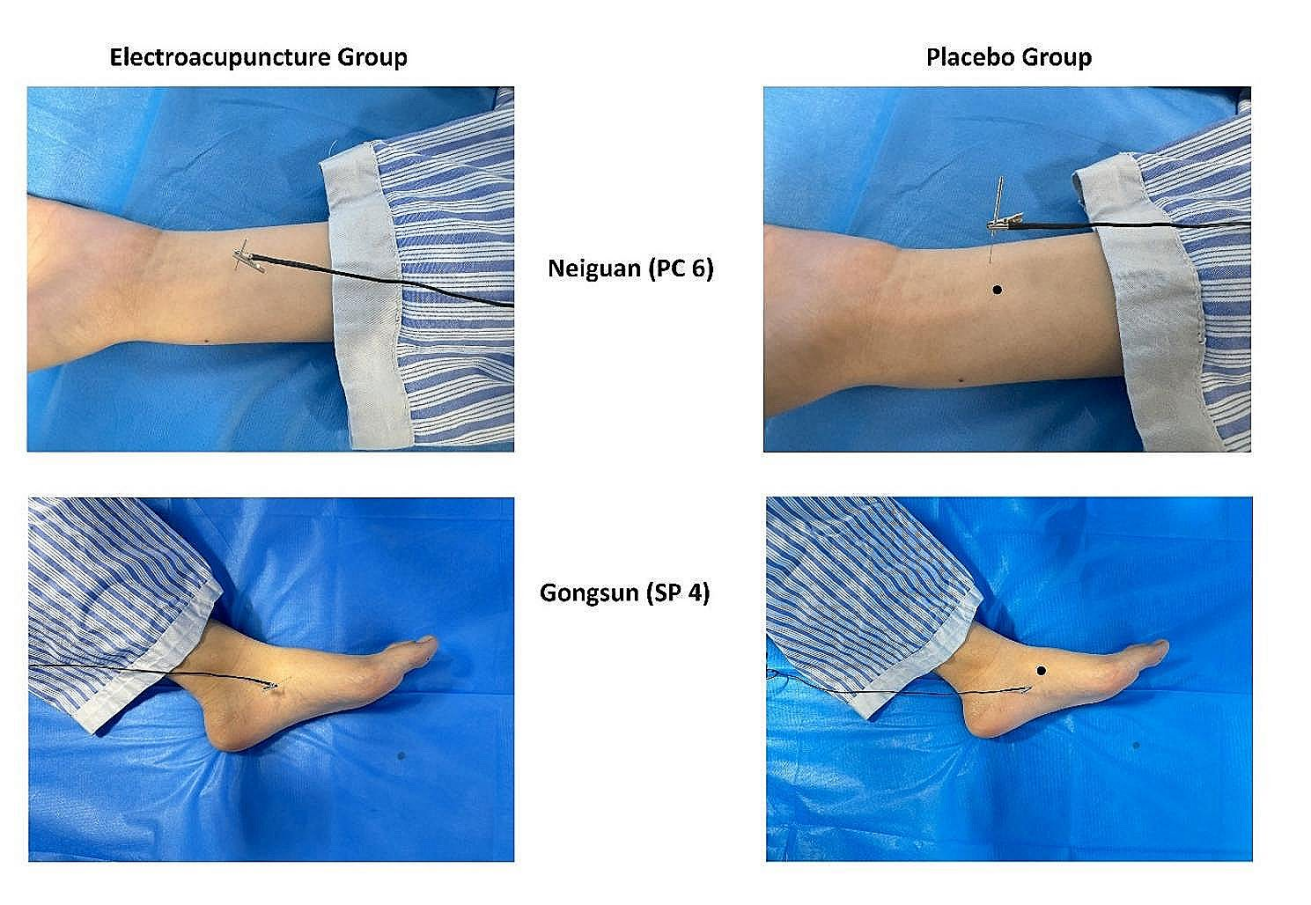
The location of Neiguan (PC 6) and Gongsun (SP 4) for EG and the respective locations for PG. The mark “•” on the placebo group indicates the original location of both acupoints

Participants in the PG will similarly receive electroacupuncture therapy every day for 5 days consecutively, from the first day post-surgery onwards, each session lasts 20 min. However, needles are applied on non-acupoints (1 cm apart from the acupoints of EG), with no needle manipulations performed(Fig. 2). To ensure the implementation of blinding, electrodes are attached, with a continuous wave of 10 Hz, but a lower current intensity of 0.1 mA.

### Outcome assessments

#### Primary outcome

First flatus time, which will be measured since the participants return to ward after surgery until the first passage of flatus. It will be recorded by doctors via inquiry of the participants themselves, the family members or the caregivers.

### Secondary outcomes

First sign of borborygmus and recovery of defecation, which will likewise be obtained through the participants themselves, the family members or the caregivers.

Laboratory tests, which blood sampling will be taken out by the nurses whereas the laboratory department will be in-charge of the handling of the blood tests and evaluation.

Self-rating anxiety scale, SAS, to measure patient’s anxiety state within the past week, with a total of 20 questions. It includes 15 positive-scored questions and 5 negative-scored questions, ranging from 1 to 4 and 4 to 1, respectively. The standard score is obtained by the sum of the raw scores, multiplied by 1.25. A higher standard score indicates a more severe level of anxiety.

Ford Insomnia Response to Stress Test, FIRST, to observe the likelihood of patients’ sleep quality being affected under different stress conditions, with a survey test consisting a total of 9 questions. Scores range from 1 to 4 based on severity, with a total score from 1 to 36. A higher score indicates that the patient’s sleep is more significantly affected by stress.

TCM rating scale of Gastrointestinal symptoms, to assess the efficacy of treatment based on the patient’s symptoms, with a scale including 32 clinically common symptoms of gastrointestinal diseases. It comes with scores of 0, 3, 5, 7 based on severity of each symptom. The total score ranges from 0 to 238. A higher score indicates a more severity of symptoms.

Gastrointestinal Symptoms Rating Scales, GSRS, to assess the patients’ gastrointestinal symptoms. This scale consists of 15 gastrointestinal symptom-related rating items, ranging from 0 to 3 based on severity, with a total score ranging from 0 to 45. A higher score indicates a more severity of gastrointestinal symptoms.

### Follow-up

The patient fills out the Quality-of-Life Questionnaire for Cancer Patients (QoL-C30) before discharge. The researcher has the patients complete the form during follow-up visits to the outpatient department at 1 month and 3 months post-surgery. If the patient doesn’t require a follow-up visit, the researcher conducts a phone call to inquire about the patient’s status.

### Participant timeline

**Table 2:**
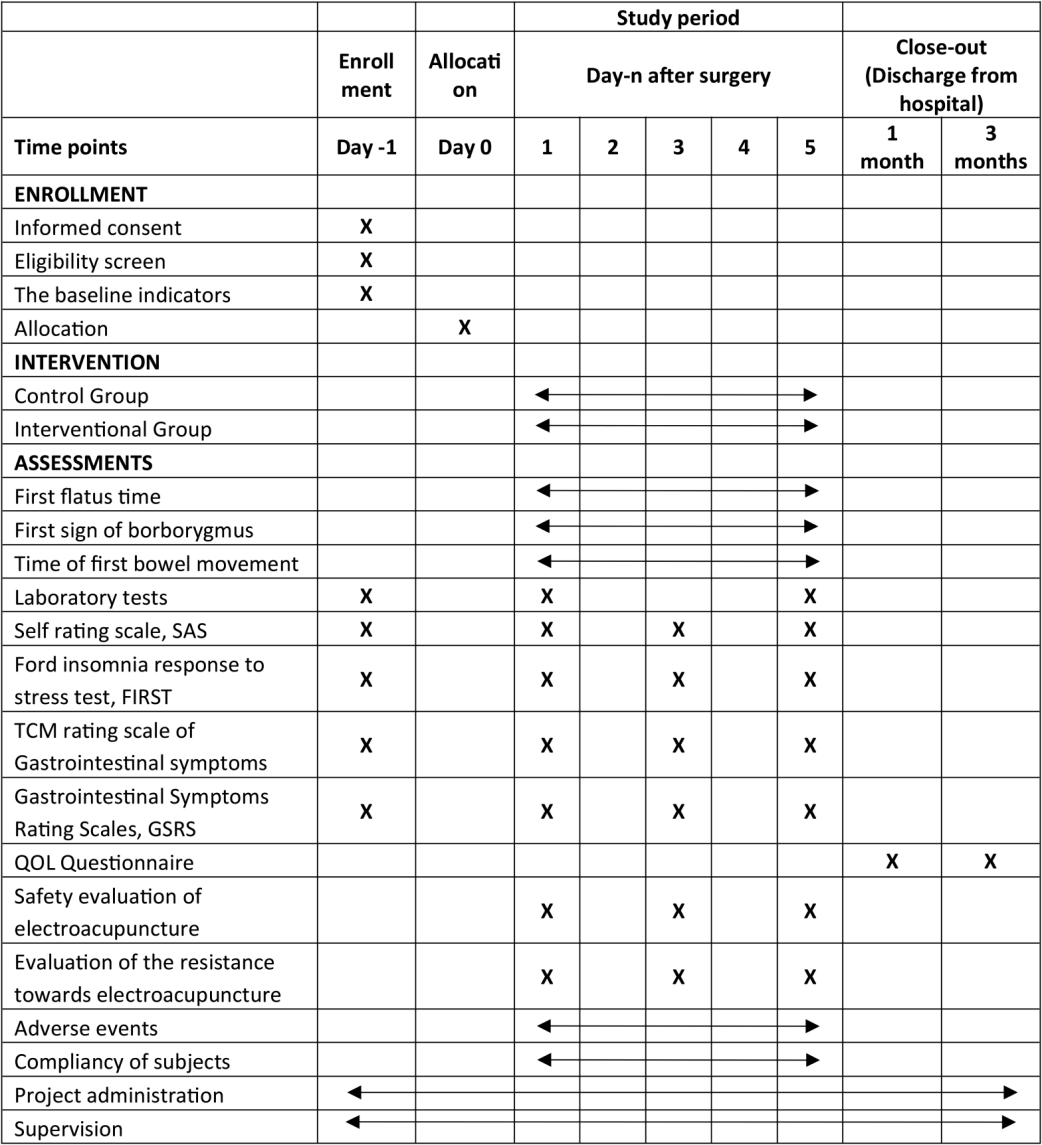
Time schedule of enrolment, interventions, and assessments

### Data collection

For the SAS, FIRST, TCM rating scale of Gastrointestinal symptoms, GSRS, safety evaluation of electroacupuncture and evaluation of the resistance towards electroacupuncture, the questionnaires will be done in Case Report Form (CRF). Three scale questionnaires shall be filled by the patients themselves or under assistance of appointed data collectors according to the participant timeline (Table 2). Participants will complete the first questionnaire before the first treatment, while the second and the third questionnaire shall be collected after the treatment on the 3rd and 5th day respectively. Data collection will be done by data collection team instead of the acupuncturists to ensure neutrality of the results.

### Data management

Data will be recorded on CRFs in paper form by the assessors. All original data will be stored in the medical record room of the Shanghai Seventh People’s Hospital for 3 years under the supervision of the Ethics Committee of Shanghai Seventh People’s Hospital affiliated to Shanghai University of Traditional Chinese Medicine.

### Statistical analyses

Data is analysed on the intention-to-treat (ITT) principle using all randomized participants. SPSS 25.0 is used to analyse data, with the statistical significance set at *P* < 0.05. Normally distributed continuous variables will be represented by mean and standard deviation (‾*x ± s*), and will be analysed using independent-samples *t*-test, whereas comparison of data within groups is done through paired *t*-test. Continuous variables that do not follow a normal distribution will be represented by median (quartiles), and will be analysed using the non-parametric test. Enumeration data will be analysed using the χ2 test. Categorical variables are analysed using a rank-sum test. Repeated measure ANOVA test will be used for statistical data recorded at multiple time points.

### Data monitoring and quality control

A Quality Control Team is instituted with the responsibility of conducting regular or ad-hoc monitoring visits and ensuring data accuracy within the research team. An independent assistant will oversee the data collection process, while the Ethics Committee of the Shanghai Seventh People’s Hospital will undertake periodic reviews. In the event of any deviations from the established protocol during monitoring, the researcher is mandated to meticulously document the deviation, outlining the time of discovery, procedural details, underlying reasons, and the corresponding corrective measures. The researcher is required to endorse this record, and the study shall be suspended.

During the statistical analysis phase, two independent study assistants are designated to input the data. They conduct self-checks to identify any discrepancies in the inputted data and promptly rectify any errors detected. Furthermore, these assistants are tasked with analysing, assessing, and reporting on the potential impact of any protocol deviations on the final data and conclusions.

### Safety evaluation

During the treatment, adverse events (AE) will be monitored through patient reports and assessments by medical workers. Adverse events include intolerance to electroacupuncture, fainting from needling, needle retention, subcutaneous bruising, local infection, etc. If a patient is intolerant to electroacupuncture, the trial can be suspended, and they may withdraw from the study. In the case of fainting, needle retention, subcutaneous bruising, follow the general procedures for the cases mentioned above, and promptly document the actions taken. Bleeding upon needle removal is common in acupuncture, and it has been proven to have therapeutic effects. Mild bleeding during acupuncture is addressed by applying pressure with a dry cotton for quick haemostasis and is not considered an adverse event (AE). For infections, consultation with a dermatologist is recommended. If serious adverse events (SAE) occur that result in disability, pose a threat to life, or lead to death, the trial should be immediately halted. Emergency measures should be taken to actively rescue the patient, stabilize vital signs, and promptly report the incident to the responsible authorities.

### Ethics and dissemination

This study protocol and informed consent materials have been approved by the Ethics Committee of Shanghai Seventh People’s Hospital affiliated to Shanghai University of Traditional Chinese Medicine; Committee approval number: 2023-7th-HIRB-078. Informed consent shall be obtained from each participant before they take part in the study. There will be prior contact with participants by the nurses or researchers before the enrolment phase, where all information about the study and the objectives, its risks and benefits will be presented. They are free to abandon the study at any time without the obligation of giving any explanation.

### Protocol amendments

If any amendments to the study are required, they shall be collaboratively agreed upon by the research team and subject to approval by the sponsor in advance. Subsequently, the research department of the Shanghai Seventh People’s Hospital will notify the China Medical Research Registration and Filing Information System of these approved changes. All modifications to the protocol will be documented, and the protocol will be accordingly updated in the clinical trial registry.

### Confidentiality

Research data will be securely stored using a study identification code assigned to each participant. The identification code list will be accessible to the research team only during the study and will be documented and safeguarded by the principal investigator upon completion of the study, in accordance with research guidelines. Patient identification details will not be disclosed in any publications.

The biological samples collected in this study will be managed by the Laboratory Department of Shanghai Seventh People’s Hospital, an entity accredited by ISO 15189. These samples will serve solely as efficacy indicators and will not be utilized for genetic or molecular analysis.

Access to the datasets generated during the current study will be available upon reasonable request through the corresponding author.

## Discussion

The acupoints Neiguan (PC 6) and Gongsun (SP 4), located at the extremities of the limbs, are more convenient compared to commonly used points such as Zusanli (ST 36), Sanyinjiao (SP 6), and Tianshu (ST 25). Patients needed not to lift their clothes, allowing researchers to operate treatment directly. This has taken care of patients who are physically weakened and sensitive to cold and wind, thereby avoiding inconveniences caused by body positioning. From the perspective of TCM, this set of acupoints not only regulates the patient’s spleen and stomach functions but also addresses issues related to the patient’s emotions and sleep quality [21–23]. During data collection of postoperative patients in the Gastrointestinal surgery department, we have discovered that patients often experience problems with emotional instability and a significant decline in sleep quality after surgery, whereas literature review indicates a certain correlation between the patient’s intestinal microbiota and anxiety [24, 25]. It is believed that patient’s emotions and sleep quality may potentially affect the recovery of gastrointestinal function, and the combination of acupoints PC 6 and SP 4 can play a role in regulating these issues.

However, there are also some shortcomings in this study. First, the sample size is restricted due to its single-centre design, which may lead to limited diversity in practice and bring risk of cofounding factors. Secondly, the treatment duration of the participants only lasts for 5 days, allowing only for the examination of short-term effects. This may not be suitable to the long-term observation of tumour patients in clinical practice. Lastly, since this study is conducted in China, where the concept of TCM and acupuncture play a significant part in the cultural context, most of the daily habits of the citizens are deeply ingrained in Chinese culture. Participants may realise that they have received placebo treatment, which could lead to a biased finding. Despite this, placebo therapy is implemented to minimise such bias.

This study is expected to contribute reliable scientific evidence for postoperative rapid recovery. It will be able to evaluate the clinical effect of electroacupuncture for post-laparoscopic patients with gastrointestinal tumour in conjunction with the ERAS concept.

### Trial status

Participants have not yet been recruited at the time of manuscript submission. Participant recruitment is expected to start in 1 January 2024, and is expected to end in 1 June 2026. Data analysis is planned to start in July 2026.

### Electronic supplementary material

Below is the link to the electronic supplementary material.

**Supplementary Material 1:** SPIRIT checklist

## Data Availability

No datasets were generated or analysed during the current study.

## Abbreviations

PG: Placebo Group
EG: Electroacupuncture Group
TCM: Traditional Chinese Medicine
PGID: Postoperative Gastrointestinal Dysfunction
ERAS: Enhanced Recovery after Surgery
RCT: Randomized Controlled Trial
PC6: Neiguan Acupoint
SP4: Gongsun Acupoint
CRF: Case Report Form
QOL: Quality-of-life
SAS: Self-rating anxiety scale
FIRST: Ford Insomnia response to stress Test
GSRS: Gastrointestinal Symptoms Rating Scales
QoL-C30: Quality-of-Life Questionnaire for Cancer Patients
